# End-users feedback and perceptions associated with the implementation of a clinical-rule based Check of Medication Appropriateness service

**DOI:** 10.1186/s12911-022-01921-7

**Published:** 2022-07-05

**Authors:** Charlotte Quintens, Willy E. Peetermans, Lorenz Van der Linden, Peter Declercq, Bart Van den Bosch, Isabel Spriet

**Affiliations:** 1 Department of Pharmaceutical and Pharmacological Sciences, KU Leuven, Louvain, Belgium; 2 Pharmacy Department, University Hospitals Leuven, Herestraat 49, 3000 Louvain, Belgium; 3 Department of Microbiology, Immunology and Transplantation, KU Leuven, Louvain, Belgium; 4 Department of General Internal Medicine, University Hospitals Leuven, Louvain, Belgium; 5 Department of Public Health and Primary Care, KU Leuven, Louvain, Belgium; 6 Department of Information Technology, University Hospitals Leuven, Louvain, Belgium

**Keywords:** Clinical pharmacy, Medication review service, End-users, Feedback, e-survey

## Abstract

**Background:**

To support appropriate prescribing hospital-wide, the ‘Check of Medication Appropriateness’ (CMA) service was implemented at the University Hospitals Leuven. The CMA concerns a clinical rule based and pharmacist-led medication review service. The aim of this study was to explore both physicians’ and pharmacists’ feedback on the optimised CMA service to further improve the service.

**Methods:**

An anonymous e-questionnaire was sent to all physicians active in the University Hospitals Leuven (n = 1631) and to all clinical pharmacists performing the CMA service (n = 16). Feedback was collected using multiple choice questions. During a 5-month period, physicians were also contacted in case of non-acceptance of recommendations to investigate barriers affecting implementation. Thematic analysis was performed and additional acceptance after telephone contact within 24 h was registered.

**Results:**

A total of 119 physicians (7.3%) and 16 pharmacists (100%) completed the e-questionnaire. The overall service was assessed as clinically relevant to highly relevant by 77.7% of physicians. The main reasons for non-acceptance of recommendations were related to workload, work environment and time constraints. About two thirds (66.3%) of initially not-accepted recommendations were accepted after phone contact. A nearly full consensus was reached among pharmacists (15/16) on the centralised CMA being complementary to current clinical pharmacy activities. Two major barriers were reported by pharmacists: (1) too limited time allocation and (2) a large number of irrelevant alerts.

**Conclusions:**

The CMA was perceived as clinically relevant by the majority of end-users. Acceptance rate of pharmaceutical recommendations was further increased by calling the physician. Increasing the specificity of clinical rules in the future is imperative.

**Supplementary Information:**

The online version contains supplementary material available at 10.1186/s12911-022-01921-7.

## Introduction

Inappropriate prescribing is common and may lead to adverse drug events, drug-related hospital (re)admissions and increased healthcare costs [1]. Several interventions have already been identified to promote appropriate prescribing and avoid the associated iatrogenic burden [2]. Software support is used increasingly to manage the large amounts of patient data, and to optimise medication safety. In this regard, major emphasis has been placed on the use of clinical decision support systems (CDSSs) [3].

CDSSs have already proven their efficacy in improving prescribing practices [3]. For instance, a Dutch interrupted time series study showed that a CDSS reduced the incidence of medication errors significantly [4]. In an RCT involving 1278 patients with chronic kidney disease, subjects were randomized to prescribers who had or did not have access to a CDSS tool. Prescribing orders were appropriately changed in 17 versus 5.7% of the time in the intervention arm and control arm, respectively (OR 1.89, 95% CI 1.45–2.47) [5]. However, alert-based CDSSs have also frequently demonstrated inconclusive effects due to low uptake and limited engagement by clinicians [3, 6]. A recent systematic review revealed that most often reported barriers for CDSS adoptation according to physicians are related to a lack of usefulness and relevance of information, and ease of use and efficiency of the system [7]. To better impact prescribing, more specific and clinically relevant approaches have been recommended. Redirecting alerts to clinical pharmacists—prior to alerting prescribers—can reduce alert fatigue and increase downstream uptake. Clinical pharmacists may be more willing to use computerised tools to optimise medication safety [8, 9].

Hence, in 2016, we designed a centralised pharmacy-led alert service to reduce potentially inappropriate prescriptions (PIPs) in inpatients at the University Hospitals Leuven (UZ Leuven): the Check of Medication Appropriateness (CMA). The CMA has been fully implemented in daily practice and currently comprises both a clinical rule-based screening for PIPs, drawing from the available information in the electronic health record (EHR), as well as a medication review performed by clinical pharmacists. Clinical rules are derived from guideline recommendations and then validated by a local multidisciplinary expert panel [10, 11]. In a first stand-alone prototype of the CMA, EHRs were screened once daily and generated alerts were listed in a non-integrated Microsoft Access database. Preliminary results showed that during an 18-month study period, 39,481 clinical rule alerts were reviewed by pharmacists for which 3205 (8.1%) recommendations were formulated. An acceptance rate by physicians of 69.4% was obtained [11].

Optimisation of the service then led to the development of the current CMA software, which is fully integrated into the EHR. This provides the opportunity to directly gather and combine information from all the databases in the EHR (e.g. medication data, laboratory values, demographics, microbiology reports, etc.). The rule-based screening now runs continuously on real-time EHR-extracted patient data and is able to discern time-dependent changes, such as those of biochemical values. Clinical rules were further reformulated and fine-tuned with the inclusion of more alert criteria [10, 12, 13]. Since January 2019, a wider range of clinical rules were implemented, specifically focusing on analgesic prescribing (e.g. screening for opioid-induced constipation) [12], antimicrobial stewardship (e.g. screening for treatment omission of *Clostridioides difficile*-associated diarrhoea) [13], intravenous (IV) to oral switch therapy [14], anticoagulant therapy (e.g. screening for inappropriate dosing of non-vitamin K antagonist oral anticoagulants) [15] and stroke prevention in atrial fibrillation patients [16]. From January 2019 to December 2020, 82,456 clinical rule alerts were reviewed and 12,688 (15.4%) recommendations were provided with an acceptance rate of 77.7%.

## Methods

### Aim

Despite the obvious increase in the recommendation rate and increase in acceptance rate, there is still ample opportunity left to further improve the performance of the service. Therefore, the goal of this study was to obtain insights in end-users perceptions and satisfaction of the overall CMA service and to identify barriers affecting physicians’ acceptance of pharmacist recommendations.

### Study design and setting

A mixed-methods cross-sectional study applying survey and interview methodologies was conducted at UZ Leuven, a 1950-bed tertiary care hospital in Belgium. The CMA is a hospital-wide, centralised clinical service, provided to all non-critically ill inpatients [11]. Funding is currently provided for a 0.6 full time equivalent (FTE) service. The CMA is performed each afternoon from Monday until Saturday by a trained clinical pharmacist. The pharmacist performs a medication review of selected drug therapies or indications, based on clinical rules which provide the pharmacist with an overview of clinical rules alerts of patients that are most at risk for predefined PIPs. Each clinical rule alert is provided with a validated flowchart (an example is provided in Additional file 1: Fig. S1) to support clinical pharmacists when performing medication reviews. For clinical relevant alerts, the pharmacist adds a note directly in the EHR addressing the treating physician based on predefined, but adaptable, recommendations. Acceptance of recommendations is assessed by reviewing the EHR.

### Data collection

The data for this study was collected in three phases. First, an anonymous e-survey was conducted to evaluate physicians’ feedback and satisfaction. The e-questionnaire was developed using Google Forms and sent via e-mail to all staff physicians and physicians in training who were active in UZ Leuven (n = 1631). The questionnaire was sent on June 24th and terminated on July 26th 2020. A reminder was sent after two weeks. The content of the survey was developed by two clinical pharmacists (CQ, IS) (Additional file 2: Table S1). Six multiple choice questions were drafted, with the aim of gathering information on: (1) knowledge of the service; (2) relevance of the overall service; (3) relevance of the recommendations for IV to oral switch; (4) relevance of the content of the clinical rules; (5) preferred communication method to provide the pharmacist recommendations and 6) the quality of the recommendations. Questions 2, 3 and 6 were scored on a 4-point Likert scale (ranging from very irrelevant to very relevant for questions 2 and 3; and from inadequate to very good for question 6). Each multiple choice question included an option to provide additional remarks.

Second, during a 5-month period (January 2021–May 2021), physicians were also contacted by phone in case of non-acceptance of pharmacist recommendations within 48 h after the CMA intervention. Physicians were questioned if the recommendation was noticed and if so, about the predominant reason and specific clinical arguments driving their decision making on the recommendation. Notes were taken during these interviews. Furthermore, for each non-accepted recommendation after 48 h, additional acceptance after telephone contact within 24 h was registered. Calling physicians and monitoring the acceptance rate was performed by a clinical pharmacist (CQ).

Third, pharmacists’ satisfaction and perceptions were also evaluated by an e-survey developed via Google Forms. The e-questionnaire was sent via e-mail on June 8th 2021 to all staff clinical pharmacists who routinely performed the CMA up to that point (n = 16). Following themes of the structured questions were defined by two clinical pharmacists (CQ, IS) (Additional file 2: Table S2): satisfaction with the service; feeling competent to perform the service; usability of the integrated worklist; usability, clarity and completeness of the flowcharts; usability of the predefined recommendations; clinical relevance of the decision rules; time allocation and overall organisation of clinical pharmacy activities in UZ Leuven. Pharmacists had the choice to complete the questionnaire anonymously or by name. Answers were collected using multiple choice questions with free-text areas for additional remarks. Pharmacists’ feedback was scored using a 4-point Likert-scale for each question (Additional file 2: Table S2).

### Analysis

Quantitative and qualitative approaches were combined for data analysis. Survey data were retrieved from Google Forms. Descriptive analyses were used to summarise the results of the multiple choice questions of both e-surveys. Categorical data were presented as counts and percentages. Thematic analysis based on an inductive approach was applied for the free-text remarks on the multiple choice questions.

Textual qualitative data gathered by telephone contact with physicians were also analysed using inductive thematic analysis. Answers were grouped and recoded independently by the primary researcher (CQ) and a senior clinical pharmacist (IS) until consensus was reached for the interpretation of all answers. Additional acceptance within 24 h after the phone contact was assessed by reviewing the EHR. Acceptance was defined as a modification of therapy (stop, start or dose correction) or a further follow-up of clinical and/or laboratory parameters.

## Results

### Prescribers’ perceptions: e-survey

Answers were retrieved from 119 (n = 1631; 7.3%) physicians, comprising 33 staff physicians and 86 physicians in training. In total, 33 different medical disciplines were represented with most of the physicians active on the general internal medicine ward (n = 15; 12.6%). One hundred physicians (84.0%) (i.e. 25/33 (75.8%) staff physicians; 75/86 (87.2%) physicians in training) indicated to be familiar with the CMA service. The following five multiple choice questions (relevance of the overall service; relevance of the recommendations for IV to oral switch; relevance of the content of the clinical rules; preferred communication method to provide the pharmacist recommendations and the quality of the recommendations) were answered by 103, 105, 116, 117 and 102 physicians, respectively.

**Fig. 1:**
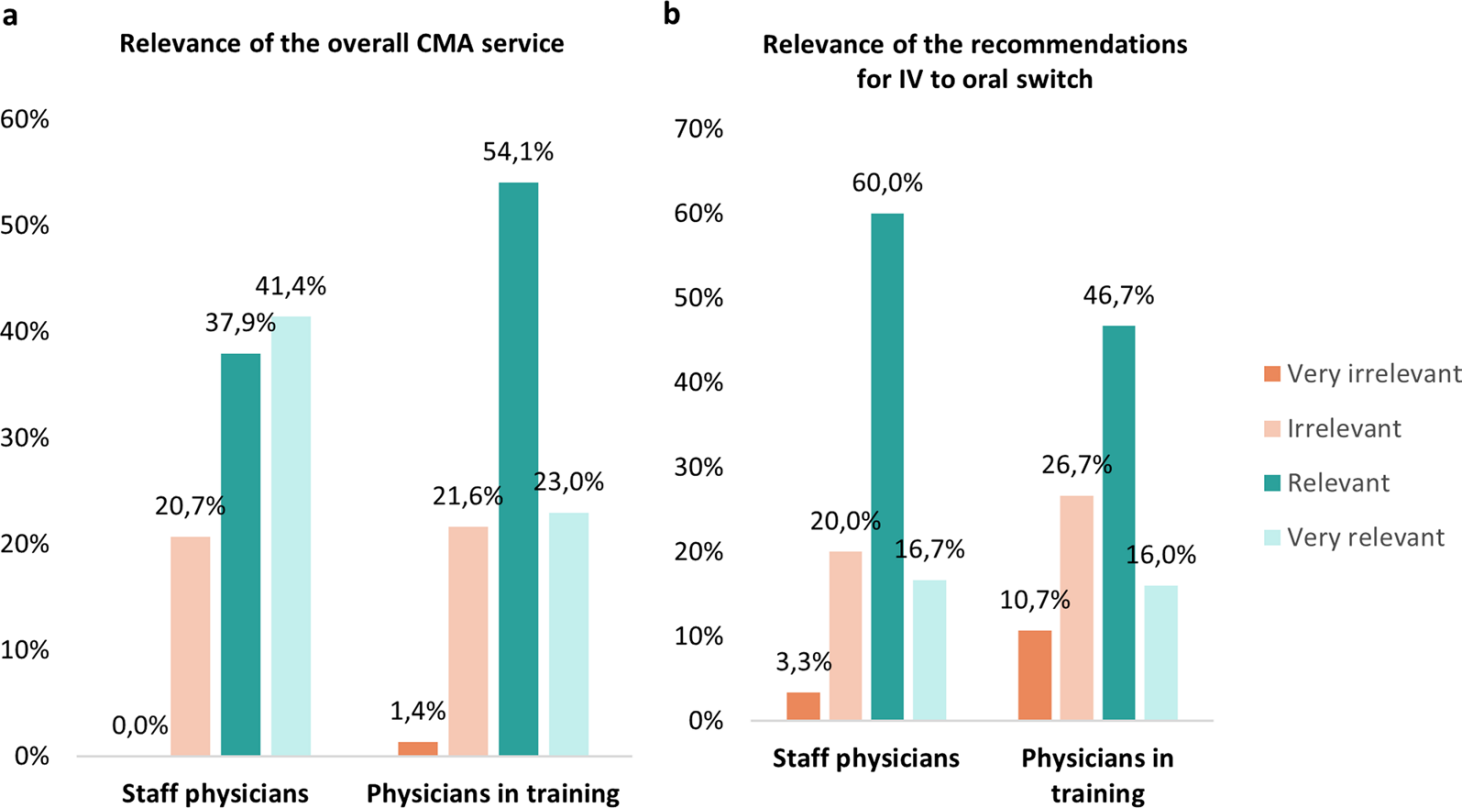
Relevance of **a** the overall CMA service (n = 103) and **b** recommendations for IV to oral switch (n = 105), according to the physician’s level of experience. CMA: check of medication appropriateness; IV: intravenous

The overall CMA service and specifically the recommendations for IV to oral switch were assessed relevant to very relevant by 77.7% (80/103) and 66.7% (70/105) of the physicians, respectively. The distribution according to physician’s level of experience is shown in Fig. 1. A main remark, mentioned by eight physicians, was that recommendations based on guidelines do not always take into account the particular clinical and medical context of the patient. Alerts for IV to oral switch therapy were rather considered ‘annoying’ by five physicians. Twenty three participants stated that there is usually a good (clinical/medical) reason to continue IV therapy. One physician, who rated the IV to oral switch service as not relevant, further indicated that such alerts are rather redundant, and that the switch may even take place fully automatically without the physician's permission.

**Fig. 2:**
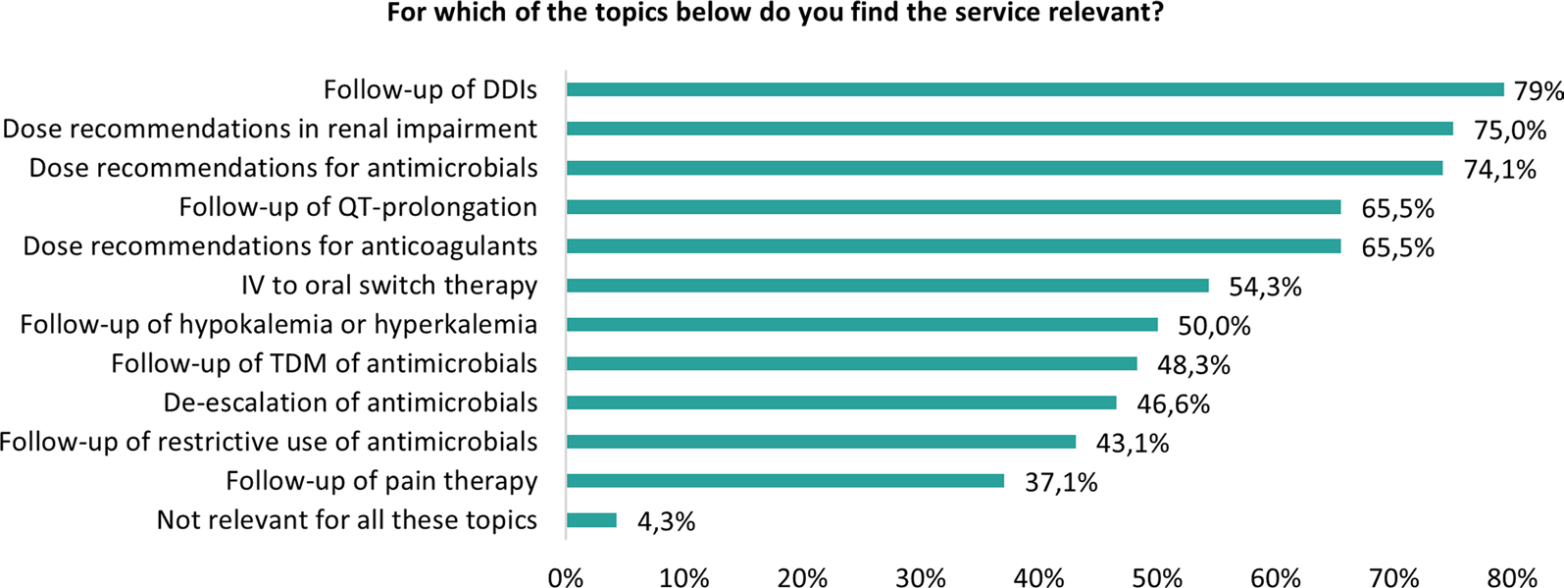
Relevance of selected topics focused on in the CMA according to the physicians (n = 116). CMA: check of medication appropriateness; DDIs: drug-drug interactions; IV: intravenous; TDM: therapeutic drug monitoring

The clinical topic which was most frequently selected as clinically relevant was the follow-up of drug-drug interactions (92/116; 79%), followed by dose recommendations in patients with abnormal kidney function (87/116; 75%) and dose recommendations for antimicrobial agents (86/116; 74.1%). Five physicians assessed the CMA as not relevant for all the proposed CMA topics (Fig. 2). Suggestions to further expand the CMA service in the near future comprised the inclusion of more clinical rules focusing on the paediatric population (n = 2), recommendations based on the STOPP/START criteria [17] (n = 1), follow-up of patients who receive total parenteral nutrition (n = 1), follow-up of pharmacological management of acute delirium [1] and recommendations on the management of arterial hypertension (n = 1).

**Fig. 3:**
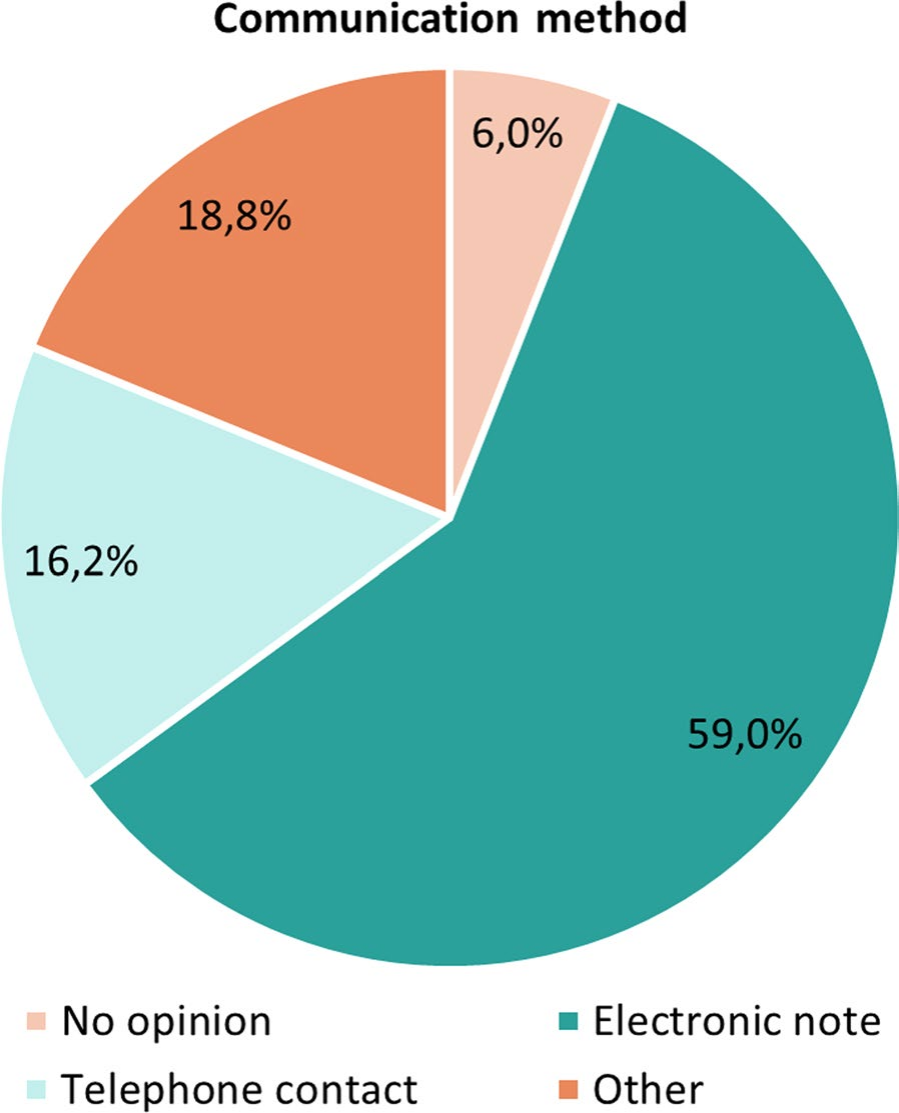
Preferred communication method to provide the pharmacist recommendations according to physicians (n = 117)

**Fig. 4:**
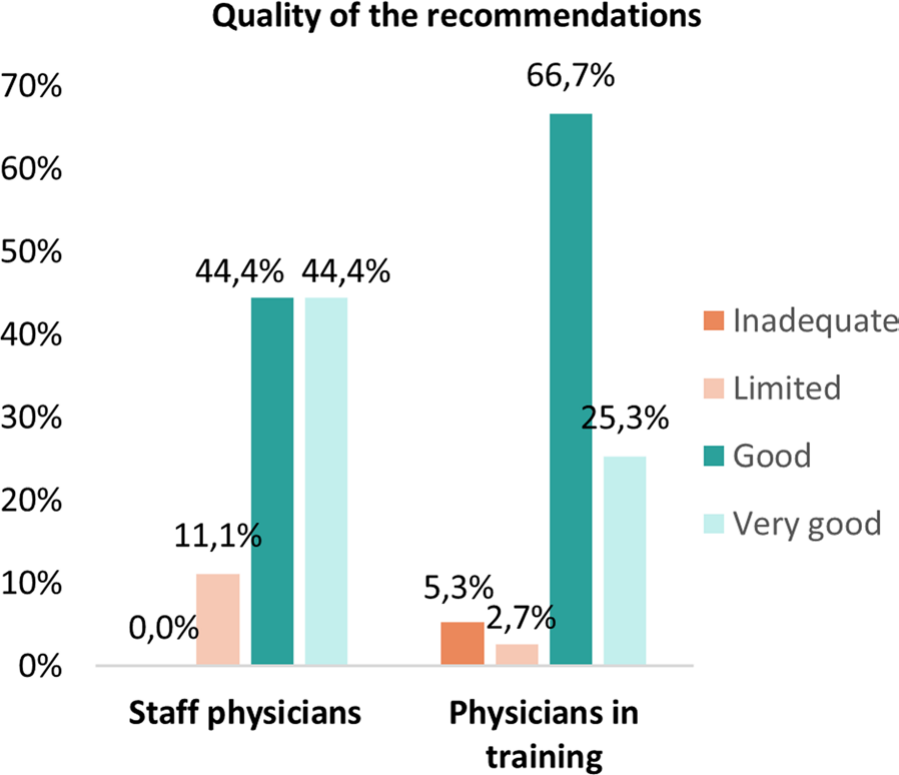
Quality of provided pharmacists recommendations according to the physician’s level of experience (n = 102)

Electronic notes were considered the preferred way of providing the recommendations by 59% (69/117) of physicians (Fig. 3) (i.e. 19/32 (59.4%) staff physicians; 50/85 (58.8%) physicians in training). Other proposals for communication included: electronic notes supplemented with telephone contact in case of severe or urgent risks (n = 17), alerts generated directly in the computerised physician order entry upon prescribing (n = 1), electronic notes with the option to remove (n = 1) and direct contact at the ward (n = 1). The quality of the pharmacist recommendations was found to be good to very good according to 91.2% (93/102) of the physicians. Figure 4 shows the response according to the physician’s level of experience.

### Prescribers’ perceptions: non-accepted recommendations

**Fig. 5:**
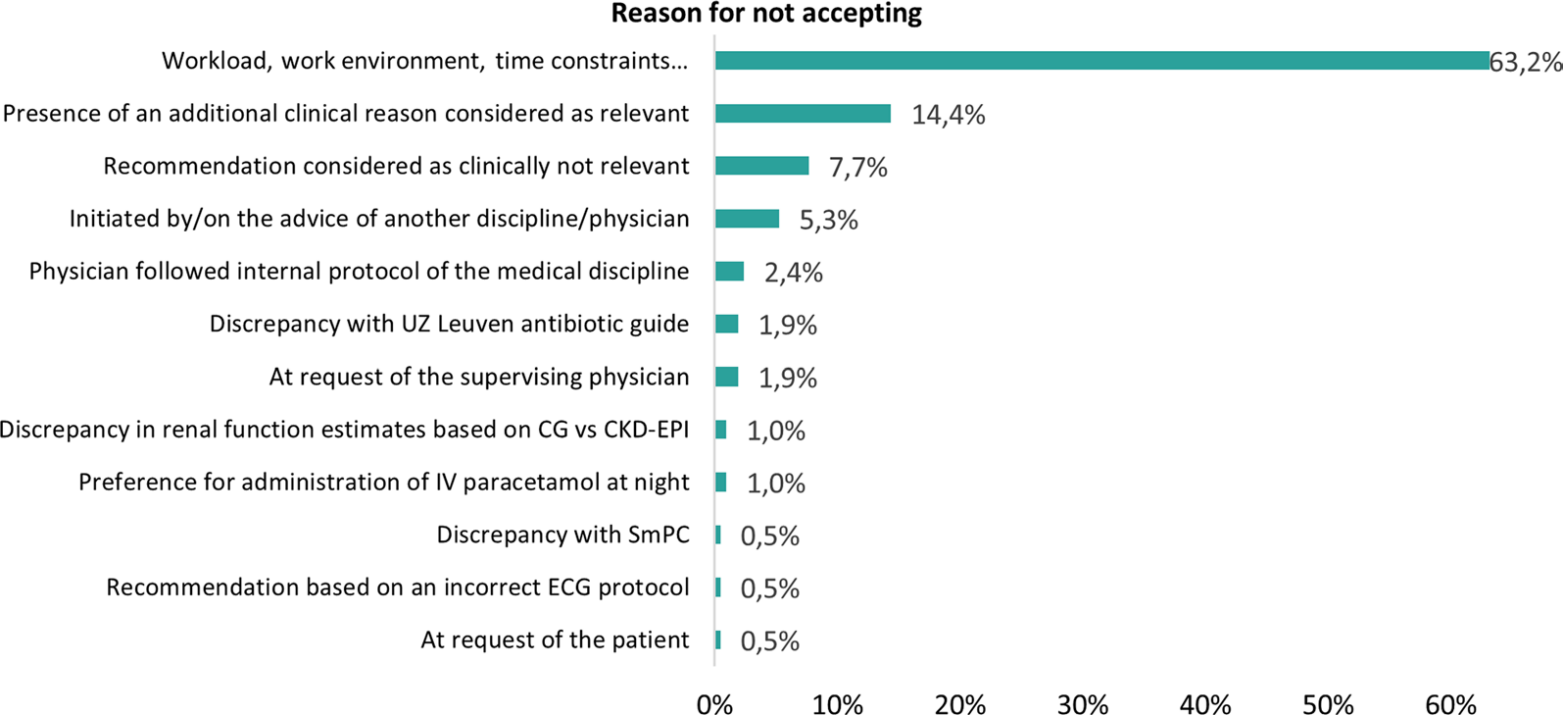
Reasons for not accepting the pharmacist recommendation (n = 209). CG: Cockcroft-Gault; CKD-EPI: Chronic Kidney Disease Epidemiology Collaboration; ECG: electrocardiogram; IV: intravenous; SmPC: summary of product characteristics; UZ Leuven: University Hospitals Leuven

During a 5-month period, treating physicians were called to discuss non-acceptance of 209 PIPs. This was limited to contact with the physicians in training as they are almost exclusively responsible for prescribing. Most phone calls were performed for persistent PIPs identified in patients admitted at the abdominal surgery ward (11.0%) and pulmonology ward (9.1%). Four barriers were predominant in influencing acceptance (Fig. 5): (1) workload, work environment and time constraints (n = 132); (2) presence of an additional clinical factor that was considered relevant (n = 30; Table 1); (3) low clinical relevance of recommendations (n = 16) and (4) reluctance towards altering prescriptions initiated by another physician (n = 11). Reasons related to the hospital environment mainly included simply not noticing the recommendation in the EHR, lack of time, new patients and forgetting to adjust therapy.

**Table 1:** Clinical reasons for non-acceptance of pharmacist recommendations mentioned by treating physicians

Recommendation (n)	Clinical reason for non-acceptance mentioned by treating physician (n)
Dose increase of anticoagulants (13)	Presence of a (temporary) bleeding^a^ (4)
	Thrombopenia (2)
	High bleeding risk (2)
	Presence of surgical drain (2)
	Acute renal failure (CrCl still above the threshold for dose reduction) (1)
	Low body weight (but still above the threshold for dose reduction) (1)
	Registered weight not corrected for presence of large abdominal cyst (1)
Dose reduction of anticoagulants (5)	Portal vein thrombosis (1)
	Transition to palliative care^a^ (1)
	Stent in superior mesenteric artery (1)
	Arteriovenous malformation (1)
	Intestinal ischemia (1)
IV to oral switch of paracetamol (4)	Difficult oral intake^a^ (2)
	Patient in a lot of pain (1)
	High fevers (1)
De-escalation of broad-spectrum antimicrobial therapy (3)	Based on clinical status (2)
	Based on history of positive cultures (1)
Dose reduction of antibiotics based on renal function (2)	*Pseudomonas aeruginosa* colonisation^a^ (1)
	Based on clinical status (1)
Dose increase of antibiotics (1)	Low body weight (1)
Switch from LMWH to oral anticoagulant (1)	Bloody wound (1)
Dose reduction of paracetamol (1)	Transition to palliative care^a^ (1)

^a^Clinical reason already included in the original algorithm of the clinical rule or the flowchart for medication review

*CrCl* creatinine clearance, *LMWH* low molecular weight heparin

**Fig. 6:**
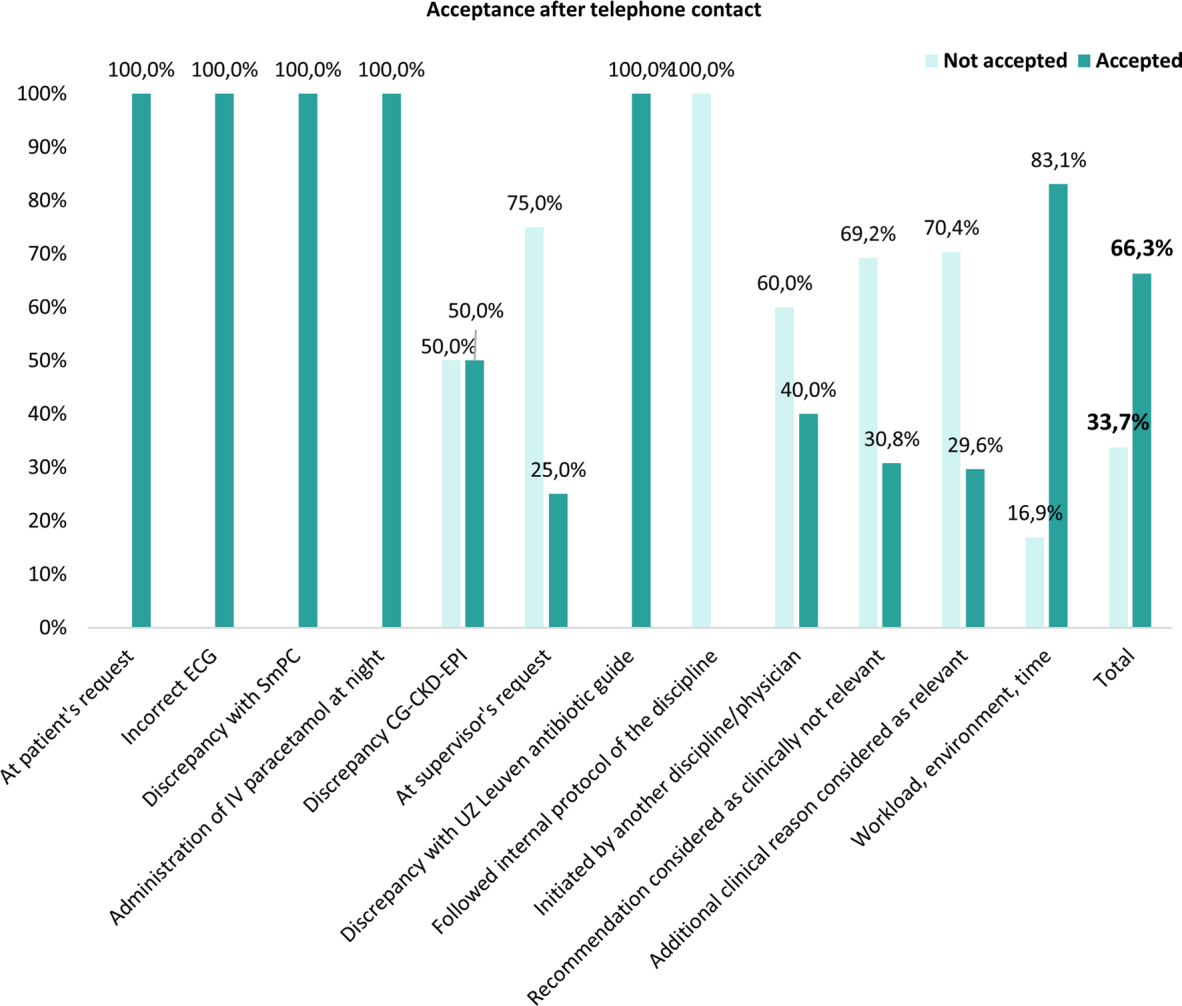
Acceptance of the recommendations by physicians after initial non-acceptance and additional telephone contact (n = 187). CG: Cockcroft-Gault; CKD-EPI: Chronic Kidney Disease Epidemiology Collaboration; ECG: electrocardiogram; IV: intravenous; SmPC: summary of product characteristics; UZ Leuven: University Hospitals Leuven

Follow-up after the phone contact revealed an additional acceptance of the CMA recommendations in 66.3% (124/187) of the persistent PIPs. The distribution of acceptance after telephone contact according to the different physician’s reason for initial non-adherence is shown in Fig. 6.

Phone calls were made for 23 non-accepted IV to oral switch recommendations. There was no clear reason for non-acceptance in 16 (69.6%) cases. In four patients there was a clinical reason that justified, according to the physician, continuation of IV paracetamol therapy (Table 1). In two patients the IV therapy was continued because they preferred to allow nurses to administer paracetamol IV overnight without disturbing the patient. For one patient, the physician in training stated that this was done specifically at the request of the supervisor, without additional information. However, additional telephone contact resulted eventually in IV to oral switch in 86.4% (19/22) of the initially non-accepted recommendations.

### Pharmacists’ perceptions: e-survey

All 16 staff clinical pharmacists completed the e-questionnaire (100%) by name. Satisfaction with the CMA service was very high: 62.5% of pharmacists were very satisfied, 31.3% were satisfied. One pharmacist (6.3%) was dissatisfied due to the high rate of irrelevant alerts.

All pharmacists indicated to feel competent when performing the CMA (37.5% very competent; 62.5% competent). Certain clinical rules were considered more complex than others, depending on the specialty of the individual pharmacist (n = 5).

Usability of the integrated CMA worklist in the EHR was overall perceived very user-friendly (75% very user-friendly; 18.8% user-friendly; 6.3% not user-friendly). All pharmacists agreed on the usability of the flowcharts (62.5% very user-friendly; 37.5% user-friendly), clarity of the flowcharts (81.3% very clear; 18.8% clear) and usability of the predefined recommendations (56.3% very user-friendly; 43.8% user-friendly). Six pharmacists stated that they usually customise the predefined recommendations based on patient-specific data to provide more individualised messages to the prescriber.

Almost all pharmacists agreed on the completeness of the flowcharts (56.3% very complete; 37.5% complete, 6.3% incomplete). The most important remark was that not all clinical questions and nuances or all clinical exceptions can be captured in a structured flowchart that is applicable for each individual patient. Consequently, in order to competently perform the CMA, pharmacists emphasised the importance of ward-based training in clinical pharmacy (n = 6).

The majority of pharmacists rated the clinical rules as clinically relevant (18.8% very relevant; 62.5% relevant; 18.8% irrelevant). Some pharmacists scored this item as less relevant because they questioned the clinical relevance of some specific rules (i.e. screening for opioid-induced constipation (n = 6), QTc prolongation (n = 3), opioid-induced nausea (n = 1), incorrect dosing of paracetamol (n = 1), overridden very severe drug-drug interactions (n = 1), intake of rivaroxaban 15 mg/20 mg without food (n = 1) and IV to oral switch therapy (n = 1)).

None of the pharmacists agreed that the time commitment of the 0.6 FTE mandate is sufficient (62.5% completely disagree; 37.5% not agree). Statements on the limited time allocation were further supplemented with remarks on the high rate of clinically irrelevant alerts (n = 4).

With the exception of a single respondent, all clinical pharmacists agreed that the centralised CMA service is complementary to the current bedside clinical pharmacy activities (37.5% completely agree; 56.3% agree; 6.3% not agree).

## Discussion

In order to evaluate and further improve the CMA service, feedback was gathered from both physicians and pharmacists. The overall service was scored positively by all respondents. Evaluation of non-accepted recommendations revealed the main reasons for non-adherence and highlighted the added value of contacting physicians by phone.

The majority of physicians assessed the overall CMA service as clinically relevant, which translated into a similar acceptance rate of pharmacists recommendations in daily practice. Furthermore, the quality of the pharmacotherapeutic recommendations, provided as an electronic note directly in the patient’s EHR, was considered as good to very good by more than 90% of physicians. Conversely, eight (6.7%) physicians specifically referred to the suboptimal wording of the recommendations, which were considered as being insufficiently tailored to the individual patient. The risk of alerts being irrelevant for the specific clinical patient context was already highlighted in other studies evaluating the uptake of alerts [18–20]. In the CMA, predefined recommendations are formulated for each clinical rule. Yet, before sending an alert to the physician, the recommendation can be adapted by adding patient-specific data to generate more individualised messages, and subsequently to improve downstream uptake. Efforts should be continued to further improve this.

The perception of the relevance of recommendations for IV to oral switch was found different according to the physician’s level of experience (Fig. 1). Twenty-three of the 35 physicians who rated the switch recommendations as irrelevant, stated that there is usually a (clinical/medical) reason to continue IV therapy. Unfortunately, the study of the non-accepted switch recommendations could only identify three specific clinical reasons. Two of these, i.e. the degree of pain and fever, are not included in the clinical rule or medication review. The development of the IV to oral switch clinical rules was however based on a definite set of 13 switch criteria, obtained by literature search and validated by a multidisciplinary expert panel [14]. Specific clinical criteria such as pain, temperature, blood leucocyte count, C-reactive protein, respiratory rate, heart rate and blood pressure were all excluded from the decision algorithm for IV to oral switch. Furthermore, we already showed the effectiveness of the switch recommendations in an interrupted time series analysis. The implementation of IV to oral switch clinical rules in the CMA showed a relative reduction of 79% (*p* < 0.0001) in residual potentially inappropriate IV prescriptions [14].

Likewise, less than 40% of physicians agreed on the relevance for follow-up of pain therapy and also fewer physicians agreed on the relevance of topics related to monitoring antimicrobial therapy. This is surprising, as this is in clear contrast to the results observed in our interrupted time series analyses evaluating the impact of clinical rules focusing on analgesic prescribing and antimicrobial stewardship. In these studies, we showed that the CMA approach reduced the number of pain-related and antimicrobial stewardship-related residual PIPs in a significant and sustained manner by 66% [12] and 86.7% [13], respectively.

In the CMA, pharmaceutical recommendations are mainly communicated to physicians via an electronic note in the EHR. For (very) severe or urgent risks, the physician is also called by phone. Based on the survey, this approach appeared to be the preferred way according to physicians. Previous studies already showed however that verbally communicated pharmaceutical recommendations are more likely to be accepted [21]. Despite the already high acceptance rate of 77.7%, we also showed an additional acceptance of 66.3% after contacting the physician by phone for initially non-accepted recommendations. This means that the overall acceptance rate could be increased from 77.7 to 92.5%. Oral communication allows the pharmacist on the one hand to alert the physician for a message that may have been overlooked or forgotten and, on the other hand, to provide more (clinical) information about the content and relevance of the advice or to gain insights in the clinical reasoning behind the non-acceptance. In the future, it is therefore recommended to systematically call the physician in case of non-adherence to improve the downstream uptake of alerts. This is further corroborated by the high percentage of non-acceptance purely as a result of workload, work environment and time constraints. Overall, four key barriers [(1) the hospital environment; (2) presence of an additional clinical factor that was considered relevant; (3) low clinical relevance of recommendations and (4) reluctance towards altering prescriptions initiated by another physician] were predominant in influencing prescribers’ acceptance of recommendations. A previous investigation by Dalton et al. already highlighted the same factors affecting prescriber implementation of SENATOR recommendations. The authors also concluded that prescribing advice should be combined with an additional face-to-face interaction with expert physicians or pharmacists to be effective [20].

The presence of an additional clinical reason considered as relevant by the physician was the second most common reason for not accepting the recommendations. This indicates that physicians may consider other factors beyond those included in the clinical rule and medication review. Consequently, some clinical rules may need to be revised, for instance by including thrombopenia and body weight when considering dose recommendations for anticoagulants and antibiotics, respectively (Table 1). On the other hand, not all of these clinical arguments were justified or based on evidence (e.g. the presence of arteriovenous malformation for dose reductions of anticoagulants). In addition, these clinical arguments might also be underreported in the EHR or even be completely missed during the review (e.g. presence of bleeding, difficulties with oral intake). This rather further supports the idea of systematically discussing the pharmaceutical recommendation with the prescriber by phone in case of non-acceptance.

Pharmacists agreed on the usefulness of the standardised flowcharts as these provide a practical guide for medication review. Importantly, since not every clinical nuance can be included in a flowchart, minimal clinical skills remain mandatory. This should be further addressed through education and training sessions. Until now, only an introductory session is provided [11]. Regularly recurring educational sessions with a focus on new rules, changes in clinical rules and complex patient cases could add value.

All pharmacists concluded that 0.6 FTE, which is currently foreseen to run the service, should be expanded in the future. Especially given the need to provide more individualised recommendations and the need to systematically call the physician in case of non-acceptance. Furthermore, a major obstacle cited by the pharmacists is the large number of false positive and thus irrelevant alerts. This problem is mainly a result of technical limitations (e.g. repeated alerts, therapy was already adjusted or stopped) and the lack of structural digitalisation of patients’ data in the EHR (e.g. health diagnoses were only mentioned in unstructured free text fields). Our ratio of recommendations (12,688 recommendations/82,456 clinical rule alerts; 15.4%) is however similar or even higher compared with results obtained in previous studies investigating pharmacy-focused alerts [22–25]. Furthermore, numerous studies have already described that patient problem lists in EHRs are often incomplete or out of date, making CDSSs less effective [26, 27]. As the pharmacists had dedicated time to perform the CMA, as opposed to alerts interrupting the many tasks of prescribing physicians, the risk of alert fatigue was expected to be lower. Although, increasing specificity of clinical rules in the future is imperative. This depends on further structural digitalisation of patients’ data in the EHR and technical operationalisation. With regard to the latter, machine learning-based methods might be a valuable option to analyse unstructured clinical data in the EHR [28, 29].

The main limitation of our study is that the surveys and interviews were not subjected to a content validation prior to sending to participants. Also, the construct and analysis was not grounded on a theoretical framework. To further assess possible additional determinants and/or challenges affecting adoptation of CMA recommendations in clinical practice, it might be recommendable to apply a theoretical adoptation model (e.g. Technology Acceptance Model [30], the Clinical Adoptation Meta-Model [31] or Human, Organization and Technology-fit (HOT-fit) model [7, 32] for analysis). Furthermore, only a sample of physicians responded, based on voluntary and anonymous participation, so self-selection bias is not excluded. This also limits the generalisability of the findings and may possibly explain the observed differences with the results of the ITS analyses. Next, only one reminder was sent to the physicians; participation rates could have been higher with one additional reminder and an extended evaluation period. Lastly, there was a year between the survey of physicians and the survey of pharmacists due to practical considerations. As a result, pharmacists have appraised a more recent version of the service. However, this may not be of great importance as very few adjustments were implemented during that year.

## Conclusions

In summary, the overall CMA service was perceived as clinically relevant by the majority of physicians. Acceptance rate of recommendations was further increased by calling the physician. Satisfaction among pharmacists was also very high. However, the current time allocation of a 0.6 FTE mandate for a large teaching hospital is too limited. Further expansion of the mandate and improving the specificity of clinical rules is therefore urgently needed. Furthermore, we need to re-evaluate and possibly adjust some clinical rules in the future.

## Supplementary Information

**Additional file 1. Figure S1.** Example of a flowchart for the clinical rule ‘Screening for vancomycin lock therapy’.

**Additional file 2. Table S1.** Physicians e-survey. **Table S2**. Pharmacists e-survey.

## Data Availability

All data generated or analysed during this current study are included in this published article.

## Abbreviations

CDSS: Clinical decision support system
CG: Cockcroft-Gault
CKD-EPI: Chronic Kidney Disease Epidemiology Collaboration
CMA: Check of Medication Appropriateness
CrCl: Creatinine clearance
DDI: Drug-drug interaction
ECG: Electrocardiogram
EHR: Electronic health record
FTE: Full time equivalent
IV: Intravenous
LMWH: Low molecular weight heparin
PIP: Potentially inappropriate prescription
SENATOR: Software ENgine for the Assessment and optimisation of drug and non-drug Therapy in Older peRsons
SmPC: Summary of product characteristics
STOPP/START: Screening Tool of Older People’s Prescriptions/Screening Tool to Alert to Right Treatment
TDM: Therapeutic drug monitoring
UZ Leuven: University Hospitals Leuven
