# Supplementary material for: End-users feedback and perceptions associated with the implementation of a clinical-rule based Check of Medication Appropriateness service
Source: BMC Med Inform Decis Mak. 2022 Jul 5;22:177. doi: 10.1186/s12911-022-01921-7 (PMC9258110; doi:10.1186/s12911-022-01921-7)

**Additional file 1**

**Figure S1.** Example of a flowchart for the clinical rule ‘Screening for vancomycin lock therapy’


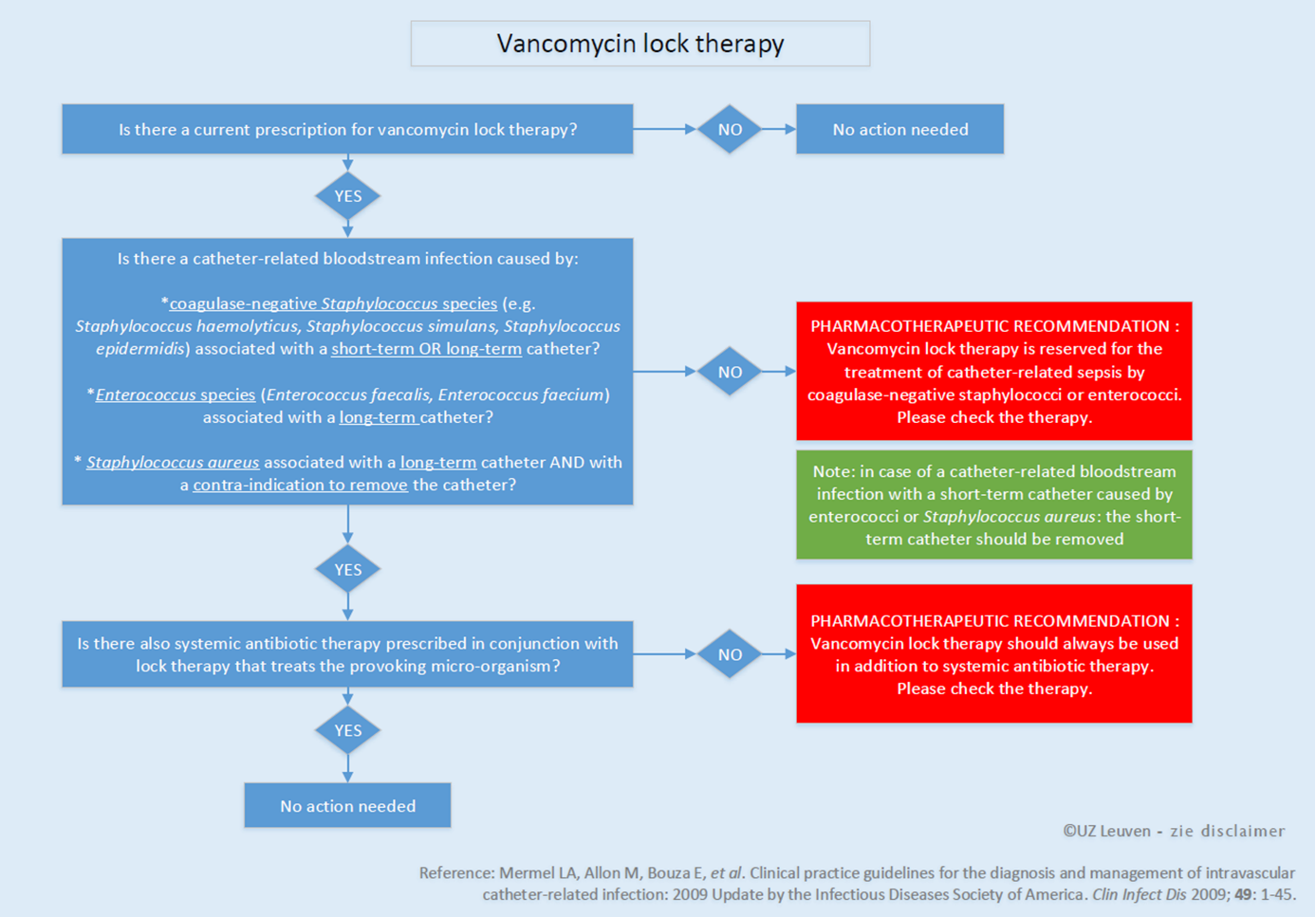

Supplement: Supplementary file 1 — Additional file 1. Figure S1. Example of a flowchart for the clinical rule ‘Screening for vancomycin lock therapy’. [file 12911_2022_1921_MOESM1_ESM.docx]
