# Supplementary material for: End-users feedback and perceptions associated with the implementation of a clinical-rule based Check of Medication Appropriateness service
Source: BMC Med Inform Decis Mak. 2022 Jul 5;22:177. doi: 10.1186/s12911-022-01921-7 (PMC9258110; doi:10.1186/s12911-022-01921-7)
Supplement: Supplementary file 2 — Additional file 2. Table S1. Physicians e-survey. Table S2. Pharmacists e-survey. [file 12911_2022_1921_MOESM2_ESM.docx]

**Additional file 2**

**Table S1.** Physicians e-survey

| **Multiple choice question 1: Do you know the ‘Check of Medication’ service?** | |
| --- | --- |
| Answer options (single select) | Yes |
|  | No |
| Additional remarks: | … |
| **Multiple choice question 2: How do you score the relevance of the overall CMA service?** | |
| Answer options (single select) | Very irrelevant |
|  | Irrelevant |
|  | Relevant |
|  | Very relevant |
| Additional remarks: | … |
| **Multiple choice question 3: How do you score the relevance of the recommendations for IV to oral switch?** | |
| Answer options (single-select) | Very irrelevant |
|  | Irrelevant |
|  | Relevant |
|  | Very relevant |
| Additional remarks: | … |
| **Multiple choice question 4: For which of the topics below do you find the service relevant (select all that are considered as relevant)?** | |
| Answer options (multi-select) | Follow-up of drug-drug interactions |
|  | Follow-up of QT-prolongation |
|  | Follow-up of hypokalemia or hyperkalemia |
|  | Follow-up of pain therapy |
|  | Follow-up of therapeutic drug monitoring of antimicrobials |
|  | Follow-up of restrictive use of antimicrobials |
|  | De-escalation of antimicrobials |
|  | Dose recommendations for antimicrobials |
|  | Dose recommendations for anticoagulants |
|  | Dose recommendations in renal impairment |
|  | IV to oral switch therapy |
|  | Relevant for all these topics |
|  | Not relevant for all these topics |
| Additional remarks and/or suggestions to further expand the CMA service: | … |
| **Multiple choice question 5: Which communication method do you prefer for providing the pharmacist recommendations?** | |
| Answer options (single-select) | Electronic note |
|  | Telephone contact |
|  | Other: … *(please specify)* |
|  | No opinion |
| Additional remarks: | … |
| **Multiple choice question 6: How do you score the quality of the provided recommendations?** | |
| Answer options (single-select) | Inadequate |
|  | Limited |
|  | Good |
|  | Very good |
| Additional remarks: | … |

CMA: Check of Medication Appropriateness; IV: intravenous

**Table S2.** Pharmacists e-survey

| **Multiple choice question 1: How satisfied are you with the overall CMA service?** | |
| --- | --- |
| Answer options (single select) | Very dissatisfied |
|  | Dissatisfied |
|  | Satisfied |
|  | Very satisfied |
| Additional remarks: | … |
| **Multiple choice question 2: How competent do you feel to perform the service?** | |
| Answer options (single select) | Very incompetent |
|  | Incompetent |
|  | Competent |
|  | Very competent |
| Additional remarks: | … |
| **Multiple choice question 3: How do you score the usability of the integrated CMA worklist in the EHR?** | |
| Answer options (single-select) | Absolutely not user-friendly |
|  | Not user-friendly |
|  | User-friendly |
|  | Very user-friendly |
| Additional remarks: | … |
| **Multiple choice question 4: How do you score the usability of the flowcharts?** | |
| Answer options (single select) | Absolutely not user-friendly |
|  | Not user-friendly |
|  | User-friendly |
|  | Very user-friendly |
| Additional remarks: | … |
| **Multiple choice question 5: How do you score the clarity of the flowcharts?** | |
| Answer options (single select) | Very unclear |
|  | Unclear |
|  | Clear |
|  | Very clear |
| Additional remarks: | … |
| **Multiple choice question 6: How do you score the completeness of the flowcharts?** | |
| Answer options (single select) | Very incomplete |
|  | Incomplete |
|  | Complete |
|  | Very complete |
| Additional remarks: | … |
| **Multiple choice question 7: How do you score the usability of the predefined recommendations?** | |
| Answer options (single-select) | Absolutely not user-friendly |
|  | Not user-friendly |
|  | User-friendly |
|  | Very user-friendly |
| Additional remarks: | … |
| **Multiple choice question 8: How do you score the clinical relevance of the clinical decision rules?** | |
| Answer options (single-select) | Very irrelevant |
|  | Irrelevant |
|  | Relevant |
|  | Very relevant |
| Additional remarks: | … |
| **Multiple choice question 9: Agree or disagree - the time commitment of the 0.6 FTE mandate is sufficient.** | |
| Answer options (single-select) | Completely disagree |
|  | Not agree |
|  | Agree |
|  | Completely agree |
| Additional remarks: | … |
| **Multiple choice question 10: Agree or disagree - the centralised CMA service is complementary to the current bedside clinical pharmacy activities in UZ Leuven.** | |
| Answer options (single-select) | Completely disagree |
|  | Not agree |
|  | Agree |
|  | Completely agree |
| Additional remarks: | … |

CMA: Check of Medication Appropriateness; EHR: electronic health record; FTE: full-time equivalent.
